# Increased Risk of Cutaneous T-Cell Lymphoma Development after Dupilumab Use for Atopic Dermatitis

**DOI:** 10.1155/2024/9924306

**Published:** 2024-08-14

**Authors:** Jenna Mandel, Jaanvi Mehta, Ramsay Hafer, Mahaa Ayub, Faria Nusrat, Henry Yang, Pierluigi Porcu, Neda Nikbakht

**Affiliations:** 1 Department of Dermatology and Cutaneous Biology, Thomas Jefferson University, Philadelphia, PA, USA; 2 Division of Hematologic Malignancies and Cellular Therapy, Department of Medical Oncology, Thomas Jefferson University, Philadelphia, PA, USA

## Abstract

There have been several reports of dupilumab use and the development of CTCL; however, the risk of CTCL development has not been adequately evaluated at the population level. The objective of this study is to determine whether dupilumab administration for AD is associated with an increased risk of developing CTCL and to identify at-risk populations within this group. This retrospective cohort study used TriNetX, a deidentified medical record database including over 107 million patients, to identify eligible patients. Treatment and control groups were evaluated for the development of CTCL. Patients of any age with a documented diagnosis of AD were included. The treatment cohort included individuals treated with dupilumab, while the control cohort included AD patients treated with alternative therapies. Selected biologics were excluded from both groups. Subgroup analyses were performed to evaluate three age groups and to identify whether the risk of CTCL development was higher within a given time frame after starting dupilumab. We identified a total of 1,181,533 patients with AD. Of these, 19,612 patients were prescribed dupilumab. Both treatment and control groups included 19,612 patients matched for age, race, and sex. The mean age was 32.3 years (*P* = 0.96), and females accounted for approximately 52% (*P* = 0.93) in both groups. Patients treated with dupilumab for AD had an increased relative risk (RR) of developing CTCL compared to those never treated with dupilumab (RR = 4.59, 95% confidence interval 2.459–8.567, *P* < 0.0001). Subgroup analysis revealed that about half of the CTCL cases after dupilumab therapy (54.5%, 30/55) occurred in patients over the age of 60 years. In contrast, all CTCL cases (100%, 12/12) within the untreated cohort were observed in individuals over the age of 60. Of the patients diagnosed with CTCL following dupilumab use, the majority (62%, 34/55) were diagnosed within the first year. Overall, we find that the use of dupilumab for treating AD is associated with an increased relative risk of developing CTCL. This risk is highest in the first year of therapy and in adult patients. These findings suggest exercising caution in treating select groups of patients with dupilumab.

## 1. Introduction

Dupilumab is a highly efective biologic therapy that blocks the alpha subunit of interleukin 4 receptor (IL-4R*α*) and disrupts T-helper 2-mediated inflammation in atopic dermatitis (AD) [1, 2]. Since dupilumab became available in the U.S. market for treatment of AD in 2017, several case series and cross-sectional studies have emerged describing an association between dupilumab use in AD patients and the development of cutaneous T-cell lymphoma (CTCL) [1–11]. Dupilumab has been demonstrated to unmask, trigger, and exacerbate CTCL for reasons not yet fully understood, although several mechanisms have been proposed. Notably, numerous reports have documented patients with AD developing the most common CTCL subtypes, such as Mycosis Fungoides (MF) or its leukemic variant Sézary Syndrome (SS), following dupilumab treatment [1–11]. Although dupilumab has also been considered a potential treatment option for extreme pruritus associated with CTCL, there are few available studies demonstrating its efficacy for this indication and those that do exist lack sufficient follow-up data [12, 13].

To date, there have been a total of 39 documented CTCL cases following dupilumab use for treatment of AD worldwide, many of which progressed rapidly to advanced-stage disease (Table 1) [1–11]. Within the past two decades, 125 cases of CTCL have been reported following the administration of various biologic therapies. Dupilumab accounts for the largest percentage (39/125; 31.2%) of CTCL cases identified after biologic therapy, followed by adalimumab (29/125; 23.2%) and etanercept (24/125; 19.2%) (Supplemental Table 1). The use of TNF-alpha inhibitors and the development of CTCL have been well described in the prior literature [14, 15]. However, despite these concerning findings, population-level analyses evaluating the risk of CTCL following dupilumab use for AD are lacking. In this study, we utilized a large population database to identify and examine all cases of cutaneous T-cell lymphoma (CTCL) following dupilumab use in AD patients within the United States (U.S.).

## 2. Methods

**FIGURE 1:**
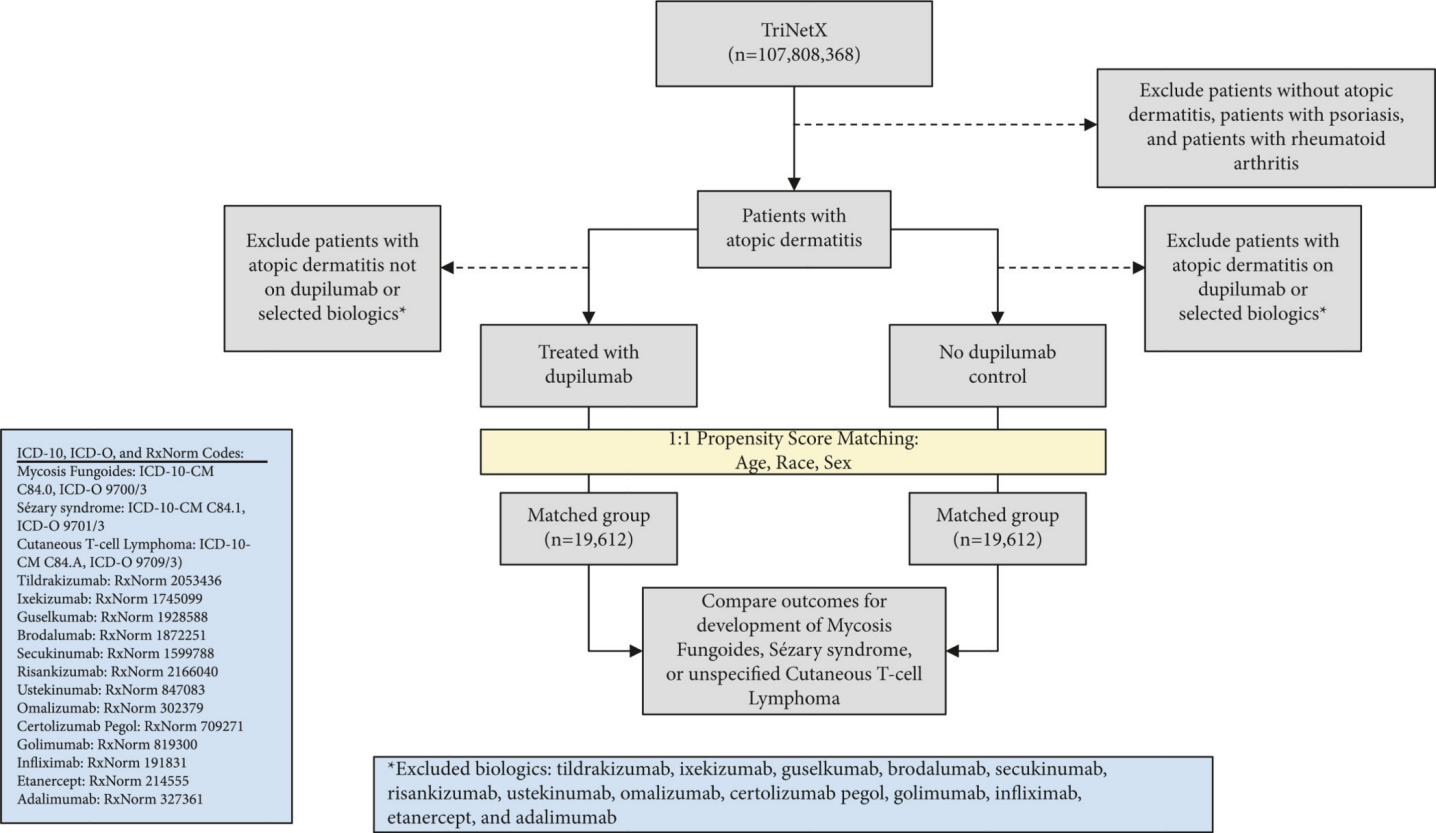
Study design.

In this retrospective cohort analysis, patients were selected utilizing TriNetX, a deidentified global health record database including over 107 million patients from 62 healthcare organizations within the U.S. collaborative network at the time of study completion in February 2024. To identify trends and inform our study design, we performed a comprehensive review of all cases of CTCL following dupilumab use documented in the literature. Our study design is depicted in Figure 1. The study was defined using ICD-10 and RxNorm codes based on medical diagnoses and prescribed drugs, respectively. Individuals diagnosed with AD who received a prescription for dupilumab were compared to those who had never received a dupilumab prescription. Patients with prior use of biologics shown in the literature to be associated with subsequent diagnosis of lymphoma were excluded [16]. Patients with inflammatory conditions that have documented associations with lymphoma, including psoriasis and rheumatoid arthritis, and the biologics indicated for the management of these conditions, were also excluded [17–19]. The complete list of excluded biologics includes ustekinumab, secukinumab, ixekizumab, brodalumab, guselkumab, risankizumab, tildrakizumab, certolizumab pegol, golimumab, infliximab, adalimumab, etanercept, and omalizumab.

After exclusion criteria were applied, a total of 1,092,798 patients were identified who had received a diagnosis of AD and who had never been treated with dupilumab or the other selected biologics. A total of 19,612 patients were identified who had received a diagnosis of AD and who had been treated with dupilumab but had never received treatment with the other selected biologics (Figure 1). Subsequently, 1 : 1 propensity score matching was applied to match cohorts for age at time of AD diagnosis, race, and biological sex, yielding two cohorts each consisting of 19,612 patients.

The risk of developing CTCL was assessed based on outcomes of Mycosis Fungoides, Sézary syndrome, and unspecified CTCL from the date of treatment initiation to the time of study completion. Subsequently, a subgroup analysis was performed to categorize patients into three age groups based on national incidence rates of CTCL: ≤40, 40–60, and ≥60 years. An additional subgroup analysis was conducted to stratify patients in the dupilumab cohort by duration of therapy. Of note, each subgroup underwent 1 : 1 propensity score matching for age at time of AD diagnosis, race, and biological sex, resulting in distinct total patient counts within each group (Table 2).

### 2.1. Statistical Analysis.

All statistical analyses were performed within the TriNetX platform. Risk assessment and *t*-test were completed for univariate analysis. Specifically, the “measures of association” functionality was selected within TriNetX for each outcome of interest. This compares the risk of developing CTCL, MF, or SS in the dupilumab cohort to the cohort never treated with dupilumab. Any outcome of CTCL, MF, or SS that occurred prior to the diagnosis of AD was excluded from the analysis. For each outcome of interest, we identified the total number of eligible patients in the cohort who met inclusion and exclusion criteria, the number of patients with the outcome, and the risk of developing said outcome relative to the cohort (i.e., risk = patients with outcome/patients in cohort). Risk ratio was also calculated (risk ratio = risk % in dupilumab cohort/risk % in no dupilumab cohort). *P* values were determined based on the risk diference (risk in dupilumab cohort–risk in no dupilumab cohort) and the associated 95% confidence intervals determined by TriNetX. *P* values less than 0.05 were considered significant.

## 3. Results

### 3.1. Comprehensive Review of All CTCL Cases Reported after Dupilumab Use.

Extensive literature review revealed a total of 39 CTCL cases diagnosed globally following the administration of dupilumab (Table 1). The majority (19/39; 48.7%) of these cases occurred in patients over the age of 60 years, while (6/39; 35.9%) occurred between the age of 40–60, and a minority (6/39; 15.4%) were identified in patients under the age of 40. Notably, the youngest patient treated with dupilumab subsequently diagnosed with CTCL was 27 years old (Supplemental Table 1). 28 (71.8%) of the reported CTCL cases were MF, and 11 (28.2%) were diagnosed as SS. Many of the patients who developed CTCL after dupilumab treatment were receiving treatment for an initial diagnosis of AD (Supplemental Table 1), and the majority (24/39; 61.5%) of these individuals had adult-onset AD, while 9 (23.1%) had a childhood history of AD, and the status of AD onset was unknown for 6 patients. On average, patients received 9.2 months of dupilumab therapy before receiving a diagnosis of CTCL.

### 3.2. Cohort Demographics.

A total of 1,181,533 patients with AD were identified within the TriNetX U.S. Collaborative network. Following application of exclusion criteria, 19,612 AD patients were identified who received dupilumab, while 1,092,798 were detected who never received dupilumab treatment (Figure 1). After 1 : 1 propensity score matching, each cohort comprised 19,612 patients matched for age at time of AD diagnosis, sex, and race. For both groups, the mean age was 32.3 years (*P* = 0.97), and females accounted for approximately 52% (Table 3). Both groups included about 50% white patients (*P* = 0.96), while the dupilumab group included 21.8% black patients and the nondupilumab group included 21.9% black patients (*P* = 0.96). Ultimately, the cohorts were well matched on the basis of age, race, and biological sex.

### 3.3. Risk of Developing CTCL in AD Patients Treated with Dupilumab Compared to Those Never Treated with Dupilumab.

In total, 55 cases of CTCL were identified in the dupilumab cohort, whereas 12 total cases of CTCL were documented in the untreated group (Table 3). The risk, or incidence, of CTCL in the dupilumab cohort was 0.28%, and the incidence of CTCL in the untreated cohort was 0.061% (Table 3). Overall, AD patients treated with dupilumab displayed an increased risk of developing CTCL compared to those never treated with dupilumab (relative risk = 4.59, 95% confidence interval 2.459–8.567, *P* < 0.0001).

### 3.4. Risk of CTCL Stratified by CTCL Subtype (Mycosis Fungoides versus Sézary Syndrome).

The majority of CTCL cases identified in the dupilumab cohort were MF (45/55, 81%), while SS accounted for only 2 cases (3.6%). The total number of MF or SS cases in the untreated cohort could not be determined due to limitations of the database for analyzing numbers less than or equal to 10.

### 3.5. Risk of CTCL Stratified by Age.

Subgroup analysis revealed diferences in the risk of CTCL based on age (Table 3). In both cohorts, patients under 40 comprised 56.1%, while those aged 40–60 comprised 16.3%, and patients over the age of 60 comprised 21.2% of the cohorts. In the dupilumab-treated cohort, 12 (21.8%) patients under the age of 40 developed CTCL, yielding a risk of 0.109%. Thirteen (23.6%) CTCL cases occurred in those aged 40–60 in the dupilumab-treated cohort, yielding a risk of 0.41%. Lastly, 30 (54.5%) patients in the dupilumab-treated cohort over the age of 60 developed CTCL, yielding a risk of 0.725% (Table 3). Although patients above the age of 60 only accounted for about a fifth of the total cohort, they experienced the most significant increased risk of CTCL development (relative risk = 2.309; 95% confidence interval 1.206–4.42) compared to the group never treated with dupilumab. In the no dupilumab cohort, all (12/12) CTCL cases identified occurred in patients above the age of 60. An additional analysis was performed to identify whether any pediatric patients developed CTCL (ages 0–18 years). No CTCL cases were identified within the pediatric age group for either cohort (Table 3).

### 3.6. Risk of CTCL Stratified by Duration of Therapy.

We performed an additional subgroup analysis within the dupilumab cohort to determine whether CTCL development was more likely to occur within the first year of dupilumab therapy, as previously reported (Table 1). Of the patients diagnosed with CTCL in the dupilumab cohort, the majority (34/55, 61.2%) were diagnosed within the first year of initiating dupilumab treatment (Table 3). After one year of dupilumab therapy, 21 patients (21/55, 38.2%) were diagnosed with CTCL (Table 3).

## 4. Discussion

We report a population-level analysis evaluating the use of dupilumab in patients with AD and the subsequent risk of developing CTCL. Overall, we find that patients with AD treated with dupilumab have a higher risk of developing CTCL compared to those never treated with dupilumab. In fact, we identify 55 cases of CTCL out of 19,612 dupilumab-treated AD patients, yielding an incidence rate of 0.28 in contrast with the reported CTCL incidence rate of 8.55 per million per year [20]. Of the CTCL cases observed in the dupilumab-treated cohort, the majority were MF and a minority were SS. Additionally, in the dupilumab-treated cohort, approximately half of the CTCL cases identified were in patients 60 and older, whereas in the untreated cohort, all CTCL cases were observed in individuals over the age of 60. Finally, most patients in the dupilumab-treated cohort developed CTCL within the first year of treatment.

Our findings reveal significant diferences in the age distribution of patients who develop CTCL in the dupilumab treated versus untreated cohorts. We interrogated three distinct age groups including patients age <40, 40–60, and ≥60 years. This design was based on the average age of CTCL diagnosis which is between 50 and 60 years, with the incidence rising in tandem with increased age [21–25]. Age distribution in the nondupilumab control group mirrored the age of CTCL onset in the general population. There were no CTCL cases in patients younger than 60 years of age in the group not receiving dupilumab. Conversely, there were a total of 25 CTCL cases identified in the dupilumab cohort for patients under 60. These findings suggest that CTCL may be detected at a younger age in AD patients treated with dupilumab.

Indeed, CTCL and AD occupy opposite ends of the spectrum for distribution at age of onset. AD tends to present in childhood, and 90% of AD cases occur within the first 5 years of life [26]. On the other hand, the incidence rate of CTCL in the pediatric population is extremely low, ranging from 0.1 to 0.3 per million persons/year compared to 17.7 to 22.9 for those over the age of 60 [27]. Although dupilumab has become a viable treatment option for children with AD as early as 6 months of age, [28] the youngest documented AD patient to receive a CTCL diagnosis following dupilumab treatment was 27 years old [29]. In our analysis, we did not identify any CTCL cases in dupilumab-treated individuals younger than 18. In the dupilumab cohort, we found that individuals with AD aged 60 and older constituted 54% of all CTCL cases and had the highest risk (0.725) of developing CTCL after treatment with dupilumab.

Various mechanisms have been suggested to explain the occurrence of CTCL in AD patients treated with dupilumab. It remains unknown if dupilumab directly triggers malignant transformation or if a pre-existing CTCL initially misdiagnosed as AD is later unmasked with dupilumab. A recent study found that 54.5% of AD patients refractory to dupilumab were subsequently diagnosed with MF upon reevaluation [30]. Patients with AD who were subsequently diagnosed with CTCL after dupilumab treatment often experienced transient symptom relief initially for an average of three months followed by disease worsening [2, 9, 30]. A speculated immunological pathway that could explain dupilumab’s role in CTCL development involves increased binding of IL-13 to its decoy receptor, IL-13R*α*2. This decoy receptor is overexpressed in malignant CD4+ T-cells and may contribute to the pathogenesis and progression of CTCL [2, 31]. Dupilumab inhibits IL-4R*α*, thereby disrupting binding of IL-4 and IL-13 to the heterodimeric receptor complex formed by IL-4R*α* and IL-13R*α*1. However, dupilumab’s blockade does not afect the binding capacity of decoy receptor IL-13R*α*2. Overall, the increased relative availability of IL-13 due to dupilumab’s blockade of IL-4R*α* may increase IL-13 binding to its IL-13R*α*2 decoy receptor, which may have downstream efects that promote CTCL progression [6, 31].

Subgroup analysis regarding duration of dupilumab treatment indicates that the risk of developing CTCL is highest within the first year after initiating dupilumab. These findings corroborate earlier studies, which collectively indicate the average duration of dupilumab therapy among AD patients who have subsequently developed CTCL is 9.2 months (Table 1). Although the exact reason for the increased risk of developing CTCL in patients treated with dupilumab is still unknown, our findings demonstrating that CTCL development is more likely to occur early in the course of dupilumab treatment align with the theory of unmasking existing MF. In other words, patients who are more susceptible to developing CTCL while receiving dupilumab for presumed AD may have had early-stage CTCL that resembled AD clinically and pathologically. Our results underscore the importance of excluding a CTCL diagnosis before starting dupilumab therapy, especially as the prevalence of AD and the use of dupilumab continue to rise.

## 5. Conclusion

Overall, our findings suggest that adult patients, specifically those without prior history of atopic conditions, should be evaluated thoroughly before initiating dupilumab. Specifically, caution should be exercised when prescribing dupilumab for individuals with later onset of AD, atypical AD presentations, or those with rapid progression of skin involvement. For patients who are refractory to dupilumab, use of skin biopsies with appropriate immunohistochemistry and clonality assays, if indicated, could be helpful in determining the correct diagnosis [30]. Moreover, for patients who develop erythroderma within one year of starting dupilumab, additional biopsies and evaluation of peripheral blood with flow cytometry may be warranted. This approach could enhance diagnostic accuracy and ensure appropriate treatment strategies. Future prospective analyses are needed to evaluate the relationship between dupilumab use in AD patients and the development of CTCL.

### 5.1. Limitations.

Instances where the recorded number of patients with a specific condition is 10 or fewer, TriNetX reports the incidence as 10, regardless of the actual incidence. Relative risks are calculated in TriNetX with the assumption that the value reported as ≤10 is 10, regardless of the true number. Our analysis using TriNetX was limited due to its retrospective nature. Given that TriNetX is a database that uses billing codes to collate data, there may have been errors within the medical records themselves.

## Supplementary Material

Supplemental Data Supplemental Table 1: provides a comprehensive summary of all documented CTCL cases identified in patients who had been treated with the selected biologics excluded from and/or relevant to this [1–11, 15, 16, 32–63] study [64–76]. (Supplementary Materials)

## Figures and Tables

**TABLE 1:** All reported cases of CTCL after dupilumab stratified by age, disease history, duration of therapy, and severity [1–11].

Age range (y)	# Of cases (%)	Avg. dupilumab duration (months)	Presentation of MF/SS (%)		Adult-onset AD (%)		
			MF	SS	Yes	No*	Unknown
<40	6 (15.4)	8.3	5 (83.3)	1 (16.7)	3 (50.0)	2 (33.3)	1 (16.7)
40–60	14 (35.9)	9.8	8 (57.1)	6 (42.9)	7 (50.0)	5 (35.7)	2 (14.3)
>60	19 (48.7)	8.4	15 (78.9)	4 (21.1)	14 (73.9)	2 (10.5)	3 (15.8)
Total	39 (100)	9.2	28 (71.8)	11 (28.2)	24 (61.5)	9 (23.1)	6 (15.4)

Avg., average; MF, mycosis fungoides; SS, Sézary syndrome

* childhood onset AD.

**Table 2:** Characteristics of study populations after matching.

	Number of patients (% within cohort)		
	Dupilumab (*n* = 19,612)	No dupilumab (*n* = 19,612)	*P* value
Age at index, mean (SD), y	32.3 (24)	32.3 (23.9)	0.9681
Sex			
Female	10,174 (51.9)	10,183 (51.9)	0.9275
Male	8,961 (45.7)	8,956 (45.7)	0.9596
Race			
White	9,808 (50.0)	9,813 (50.0)	0.9597
Black	4,285 (21.8)	4,289 (21.9)	0.9610
Asian	1,606 (8.19)	1,613 (8.23)	0.8975
Unknown race	1,045 (5.33)	1,038 (5.29)	0.8748

**Table 3:** Risk of CTCL after dupilumab use for atopic dermatitis by subgroup.

	Dupilumab			No dupilumab		
	Cohort size (% total)	CTCL cases (%risk)	% Of all CTCL cases	CTCL cases (% risk)	% Of all CTCL cases	*P* value
Total	19,612 (100)	55 (0.281)	100	12 (0.061)	100	<0.0001
CTCL subtype						
Mycosis fungoides		45 (0.229)	81.8	Unk	—	—
Sézary syndrome		2 (0.010)	3.6	Unk	—	—
Age group						
<40*	11,001 (56.1)	12 (0.109)	21.8	**	0	—
40–60	3,190 (16.3)	13 (0.41)	23.6	**	0	—
>60	4,167 (21.2)	30 (0.725)	54.5	12 (0.287)	100	0.0093
Time to CTCL development since treatment initiation						
Within first year		34	61.2	N/A	N/A	N/A
After first year		21	38.2	N/A	N/A	N/A

* All cases were identified in patients above 18 in this subgroup. There were 0 patients who developed CTCL in either cohort under 18.

** Numbers reported as ≤10 in TriNetX indicate an unreported, unknown value that is between 0 and 10. In this table, it can be deduced that the true values for those reported as ≤10 are in fact 0. However, percent risk is calculated with the assumption that this unknown value is 10. Unk; unknown. The symbols–indicate a value was not able to be calculated. N/A: not applicable.

## Data Availability

All relevant data obtained from TriNetX are reported in Table 3. The study may be reproduced using the billing codes and study design outline in our methods and Figure 1.
