# Supplementary material for: Increased Risk of Cutaneous T-Cell Lymphoma Development after Dupilumab Use for Atopic Dermatitis
Source: Dermatol Ther. Author manuscript; Available in PMC 2024 Dec 12. (PMC11635927; doi:10.1155/2024/9924306)
Supplement: Supplemental Data — Supplemental Table 1: provides a comprehensive summary of all documented CTCL cases identified in patients who had been treated with the selected biologics excluded from and/or relevant to this [1–11, 15, 16, 32–63] study [64–76]. (Supplementary Materials) [file NIHMS2039673-supplement-Supplemental_Data.pdf]

Author	Age (yr) / Sex	Treatment Duration (months)	CTCL	Initial diagnosis	Other Biologics used	Systematic Review
**Adalimumab (n=29)**						
Dalle et al., 2005[32]	40 M	7	MF	Ankylosing spondylitis		Schaefer et al., 2023[16]
Bittencourt et al., 2013[33]	48 F	27	Chronic ATLL	Spondylarthritis; childhood eczema**;** uveitis, bilateral episcleritis		Schaefer et al., 2023
D'Haens et al., 2018[34]	29 M	unk	MF	Crohn's disease		Schaefer et al., 2023
Martinez-Escala et al., 2018[15]	69 F	1	CTCL NOS	psoriasis		Schaefer et al., 2023
Martinez-Escala et al., 2018	63 M	1	CTCL NOS	Crohn's disease and psoriasis		Schaefer et al., 2023
Martinez-Escala et al., 2018	46 F	8	CTCL NOS	Crohn's Disease		Schaefer et al., 2023
Martinez-Escala et al., 2018	72 M	unk	MF	RA and dermatitis	Etanercept, infliximab	Schaefer et al., 2023
Martinez-Escala et al., 2018	29 F	unk	MF	psoriasis	ustekinumab	Schaefer et al., 2023
Martinez-Escala et al., 2018	21 M	20	MF	Psoriasis		Schaefer et al., 2023
Martinez-Escala et al., 2018	64 F	unk	MF	erythroderma	infliximab	Schaefer et al., 2023
Martinez-Escala et al., 2018	58 M	4	MF	psoriasis		Schaefer et al., 2023
Martinez-Escala et al., 2018	49 M	24	MF	psoriasis		Schaefer et al., 2023
Martinez-Escala et al., 2018	75 M	unk	MF	Erythroderma		Schaefer et al., 2023
Martinez-Escala et al., 2018	72 F	unk	MF	Eczema vs psoriasis	etanercept	Schaefer et al., 2023
Martinez-Escala et al., 2018	27 M	unk	PCAEC-TCL	Psoriatic arthritis	ustekinumab, infliximab	Schaefer et al., 2023
Martinez-Escala et al., 2018	64 M	unk	SS	psoriasis	ustekinumab	Schaefer et al., 2023
Martinez-Escala et al., 2018	40 M	unk	SS	psoriasis		Schaefer et al., 2023
Martinez-Escala et al., 2018	66 F	unk	MF	Chronic atopic derm	Dupilumab	Schaefer et al., 2023
Foo et al., 2016[35]	70	unk	MF/CTCL	psoriasis	infliximab	Biondo et al., 2019 [36]
Nikolaou et al., 2015[37]	73 M	4	MF/CTCL	psoriasis		Biondo et al., 2019
Nikolaou et al., 2015	44 F	12	MF	psoriasis	Etanercept	Biondo et al., 2019
Nikolaou et al., 2015	68 M	15	MF/CTCL	psoriasis		Biondo et al., 2019
Fensterfeifer et al., 2022[38]		unk	Primary cutaneous anaplastic large cell	psoriasis		Davis et al., 2024[14]
Diakomopoulos et al., 2021[39]	65	unk	MF	psoriasis	Secukinumab, ustekinumab	Davis et al., 2024
Rohl et al., 2018[40]	52	1.5	MF	psoriasis		Davis et al., 2024
Jacks et al., 2014[41]	35 F	2	PCAEC-TCL	psoriasis		Davis et al., 2024
Alberdi et al., 2015[42]	43 M	12	MF	psoriasis		Davis et al., 2024
Amitay-Laish et al., 2023[43]	74 M	unk	MF	psoriasis	Etanercept,golimumab	Amitay-Laish et al., 2023[43]
Amitay-Laish et al., 2023[43]	72 M	unk	MF	RA	etanercept	Amitay-Laish et al., 2023[43]
**Secukinumab (n=6)**						
De et al., 2017[44]	43 **M**	1.2	MF/CTCL	psoriasis	Etancercept	Biondo et al., 2019
Yoo et al., 2019[45]	79 **F**	3	MF/CTCL	psoriasis		Biondo et al., 2019
Yoo et al., 2019	71 M	2	MF/CTCL	psoriasis		Biondo et al., 2019

Sokumbi et al., 2021[29]	74 M	unk	MF	Chronic dermatitis	dupilumab	Schaefer et al., 2023
Amitay-Laish et al., 2020	34 F	16	MF	IBD	infliximab	Amitay-Laish et al., 2020
Diakomopoulos A et al. 2021[39]	65 M	unk	MF	Refractory psoriasis	Adalimumab, ustekinumab	
**Ustekinumab (n=3)**						
Papp et al., 2013[46]	unk	unk	CTCL NOS	psoriasis		Schaefer et al., 2023
Martinez-Escala et al., 2018[15]	29 F	unk	MF (FMF)	Psoriasis	adalimumab	Schaefer et al., 2023
Martinez-Escala et al., 2018	64 M	24	SS	Psoriasis (4 mo)	adalimumab	Schaefer et al., 2023
**Ixekizumab (n=2)**						
Partarrieu-Mejias et al., 2019[47]	53 F	unk	MF	psoriasis	Etancercept	Schaefer et al., 2023
Blanchard et al., 2024[48]	77 F	7	MF	psoriasis		
**Omalizumab (n=2)**						
Sokumbi et al., 2021[29]	73 M	unk	MF	Chronic dermatitis	Dupilumab	Schaefer et al., 2023
Jiang et al., 2024[49]	60 F	3	MF	Allergic contact dermatitis/ angioedema	Dupilumab	
**Risankizumab (n=1)**						
Fahmy et al., 2023[50]	54 M	4	MF	Psoriasis		
**Infliximab (n=19)**						
Mahe et al., 2003[51]	47 M	3	Primary cutaneous CD30+ T cell lymphoma	Psoriasis		Schaefer et al., 2023
Adams et al., 2004[52]	81 F	4	ALCL	Crohn's disease		Schaefer et al., 2023
Berthelot et al., 2006[53]	54 M	0.3	MF	Plaque psoriasis vulgaris	Efalizumab 12 months	Schaefer et al., 2023
Berthelot et al., 2006	64 M	4	SS	Nonspecific eczematous skin rashes and rheumatoid arthritis		Schaefer et al., 2023
Dauendorffer et al., 2007[54]	75 M	17	SS	Ankylosing spondylitis		Schaefer et al., 2023
Lees et al., 2009[55]	52 M	Single dose	CTCL NOS	Celiac disease		Schaefer et al., 2023
Suga et al., 2014[56]	60 F	1	MF	Psoriasis		Schaefer et al., 2023
Martinez-Escala et al., 2018	72 M	unk	MF	Dermatitis	Adalimumab,Etanercept	Schaefer et al., 2023
Martinez-Escala et al., 2018	51 F	unk	MF	Eczema (early childhood)		Schaefer et al., 2023
Martinez-Escala et al., 2018	42 M	1	PCGD-TCL	Sarcoidosis		Schaefer et al., 2023
Yasuda et al., 2021[57]	46 M	84	MF	Ulcerative colitis		Schaefer et al., 2023
Foo et al., 2016[35]	70	unk	MF	Psoriasis		Biondo et al., 2019

Nikolaou et al., 2015	71 M	2	MF	Psoriasis		Biondo et al., 2019
Nikolaou et al., 2015	67 M	3	MF	Psoriasis		Biondo et al., 2019
Nikolaou et al., 2015	73 M	5	MF	Psoriasis		Biondo et al., 2019
Amitay-Laish et al., 2020	34 F	unk	MF	IBD		Amitay-Laish et al., 2020
Sanli et al., 2007	32 M	36	MF	Ankylosing spondylitis		
Nikolaou et al., 2015[37]	51M	52	MF	psoriasis	Etanercept	Davis et al., 2024
**Etanercept (n=24)**						
Adams et al., 2004[52]	69 M	18	SS	Psoriatic arthritis		Schaefer et al., 2023
Koens et al., 2009[58]	67 M	36	PCGDTCL	Rheumatoid arthritis		Schaefer et al., 2023
Lafaille et al., 2009[59]	47 M	unk	MF	HLA B27 positive oligoarticular arthritis		Schaefer et al., 2023
Michot et al., 2009[60]	50 F	72	SPTCL	Rheumatoid arthritis		Schaefer et al., 2023
Ma et al., 2016[61]	62 M	3	PCSM-TCL	Rheumatoid arthritis		Schaefer et al., 2023
Martinez Escala et al., 2018	72 M	unk	Folliculotropic MF	Rheumatoid arthritis and dermatitis	Adalimumab,infliximab	Schaefer et al., 2023
Martinez Escala et al., 2018	76 F	6	Folliculotropic MF	Psoriasis		Schaefer et al., 2023
Martinez Escala et al., 2018	63 M	2	Folliculotropic MF	Psoriasis		Schaefer et al., 2023
Martinez Escala et al., 2018	72 F	12	MF	Rheumatoid arthritis		Schaefer et al., 2023
Martinez Escala et al., 2018	72 F	unk	MF LCT	Eczema vs psoriasis	Adalimumab	Schaefer et al., 2023
Martinez Escala et al., 2018	41 M	8	MF LyP overlap	Erythroderma		Schaefer et al., 2023
Partarrieu-Mejias et al., 2019[47]	53 F	unk	MF	Psoriasis	Ixekizumab	Schaefer et al., 2023
Partarrieu-Mejias et al., 2019	52 M	9	MF	Psoriasis		Schaefer et al., 2023
Amitay-Laish et al., 2023[43]	74 M	unk	MF	psoriasis	Adalimumab,golimumab	Amitay-Laish et al., 2023[43]
Amitay-Laish et al., 2023[43]	72 M	unk	MF	RA	adalimumab	Amitay-Laish et al., 2023[43]
Nikolaou et al., 2015[37]	73 F	12	MF/CTCL	psoriasis		Biondo et al., 2019
Schmidt et al., 2005[62]	72 M	10	MF/CTCL	psoriasis		Biondo et al., 2019
De A et al., 2017[44]	43 M	3	MF/CTCL	psoriasis		Biondo et al., 2019
Nikolaou et al., 2015[37]	51 M	24	MF	psoriasis	Infliximab	Davis et al., 2024
Nikolaou et al., 2015	44 F	12	MF	RA and psoriasis	Adalimumab	Davis et al., 2024
Moka et al., 2018	70 M	36	MF	psoriasis		Davis et al., 2024
Visentainer et al., 2018[63]	43 F	2	MF	psoriasis		Davis et al., 2024
Quereux et al., 2009[64]	36 M	3	Cutaneous pleomorphic T cell lymphoma	psoriasis		

Chuang et al., 2008[65]	61 F	6	MF	RA	
**Dupilumab (n=39)**					
Hamp et al., 2023[1]	76 M	12	MF	AD	Hamp et al., 2023
Hamp et al., 2023	36 M	2	MF	AD	Hamp et al., 2023
Hamp et al., 2023	68 F	11	MF	AD	Hamp et al., 2023
Hamp et al., 2023	59 M	unk	SS	AD	Hamp et al., 2023
Hamp et al., 2023	77 M	unk	SS	AD	
Hsieh et al., 2023[7]	34 M	4	MF	AD	
Toker et al., 2023[10]	65 M	6	CD30+ MF	AD	
Hashimoto 2022[6]	47 F	1 injection	MF	AD	
Park et al., 2022[2]	72 M	2.25	MF		
Poyner et al., 2022[66]	60 M	2.25	MF	AD	
Ahatov et al., 2022[3]	62 F	18	MF		
Buffon et al., 2023[4]	55 M	19	MF	AD	
Buffon et al., 2023	85 M	11	SS	AD	
Di Yan et al., 2023[11]	79 F	unk	MF	AD	
Ayasse et al., 2020[67]	40 F	13	MF	AD	Park et al., 2023
Chiba et al., 2019[68]	58 M	unk	MF	AD	Park et al., 2023
Espinosa et al., 2020[69]	64 M	8	CTCL NOS	AD	Park et al., 2023
Espinosa et al., 2020	72 M	4	MF	AD	Park et al., 2023
Espinosa et al., 2020	59 F	27	MF	AD	Park et al., 2023
Espinosa et al., 2020	40 F	15	MF	AD	Park et al., 2023
Hollins et al., 2020[70]	61 M	unk	MF	Nonspecific eczematous dermatitis, lichen simplex chronicus	Park et al., 2023
Hollins et al., 2020	52 M	unk	MF	Psoriasiform dermatitis	Park et al., 2023
Hollins et al., 2020	60 F	unk	MF	AD vs psoriasis vs lichenoid dermatitis	Park et al., 2023
Lazaridou et al., 2020[71]	37 F	2	SS	AD	Park et al., 2023
Miyashiro et al., 2020[72]	51 F	unk	MF	AD	Park et al., 2023
Newsom et al., 2021[73]	48 F	5	MF	AD	Park et al., 2023
Newsom et al., 2021	55 M	6	MF	AD	Park et al., 2023
Poyner et al., 2019[74]	60 M	2.25	MF	AD	Park et al., 2023
Russomanno et al., 2020[75]	43 M	2	MF	AD	Park et al., 2023
Sokumbi et al., 2021[29]	66 F	1.2	MF	dermatitis	Park et al., 2023
Sokumbi et al., 2021	65 F	2.5	MF	AD, idiopathic eosinophilia	Park et al., 2023
Sokumbi et al., 2021	74 M	24	MF	AD	Park et al., 2023
Sokumbi et al., 2021	73 M	14	MF	Chronic dermatitis	Park et al., 2023
Sokumbi et al., 2021	74 M	1	MF	chronic dermatitis	Park et al., 2023
Sokumbi et al., 2021	44 F	12	MF	AD	Park et al., 2023
Sokumbi et al., 2021	27 M	14	MF	AD, ACD	Park et al., 2023
Tran et al., 2020[76]	64 M	unk	unk	AD	Park et al., 2023

Umemoto et al., 2020[77]	48 F	2	unk	AD		Park et al., 2023
Jiang et al., 2024[49]	60 F	4	MF	Allergic contact dermatitis/ angioedema	omalizumab	

Supplemental Table 1 abbreviations: CTCL, Cutaneous T-cell lymphoma; AD, atopic dermatitis; RA, rheumatoid arthritis; IBD, inflammatory bowel disease; MF, mycosis fungoides; SS, Sézary Syndrome; PCAEC-TCL Primary cutaneous aggressive epidermotropic CD8+ T-cell lymphoma; PCGDTCL, primary cutaneous gamma-delta T-cell lymphoma; CTCL NOS, CTCL not otherwise specified; ALCL, anaplastic large cell lymphoma; LyP, lymphomatoid papulosis; unk, unknown.
